# Repositioning of Lansoprazole as a Protective Agent Against Cisplatin-Induced Ototoxicity

**DOI:** 10.3389/fphar.2022.896760

**Published:** 2022-07-15

**Authors:** Eri Wakai, Kenji Ikemura, Toshiro Mizuno, Kazuhiko Takeuchi, Satoshi Tamaru, Masahiro Okuda, Yuhei Nishimura

**Affiliations:** ^1^ Department of Integrative Pharmacology, Mie University Graduate School of Medicine, Tsu, Japan; ^2^ Department of Pharmacy, Osaka University Hospital, Suita, Japan; ^3^ Department of Medical Oncology, Mie University Graduate School of Medicine, Tsu, Japan; ^4^ Department of Otorhinolaryngology-Head and Neck Surgery, Mie University Graduate School of Medicine, Tsu, Japan; ^5^ Clinical Research Support Center, Mie University Hospital, Tsu, Japan

**Keywords:** cisplatin, ototoxicity, proton pump inhibitor, organic cation transporter 2, zebrafish, genome editing, adverse event, electronic health record

## Abstract

Cisplatin (CDDP) is a well-known chemotherapeutic drug approved for various cancers. However, CDDP accumulates in the inner ear cochlea via organic cation transporter 2 (OCT2) and causes ototoxicity, which is a major clinical limitation. Since lansoprazole (LPZ), a proton pump inhibitor, is known to inhibit OCT2-mediated transport of CDDP, we hypothesized that LPZ might ameliorate CDDP-induced ototoxicity (CIO). To test this hypothesis, we utilized *in vivo* fluorescence imaging of zebrafish sensory hair cells. The fluorescence signals in hair cells in zebrafish treated with CDDP dose-dependently decreased. Co-treatment with LPZ significantly suppressed the decrease of fluorescence signals in zebrafish treated with CDDP. Knockout of a zebrafish homolog of *OCT2* also ameliorated the reduction of fluorescence signals in hair cells in zebrafish treated with CDDP. These *in vivo* studies suggest that CDDP damages the hair cells of zebrafish through oct2-mediated accumulation and that LPZ protects against CIO, possibly inhibiting the entry of CDDP into the hair cells via oct2. We also evaluated the otoprotective effect of LPZ using a public database containing adverse event reports. The analysis revealed that the incidence rate of CIO was significantly decreased in patients treated with LPZ. We then retrospectively analyzed the medical records of Mie University Hospital to examine the otoprotective effect of LPZ. The incidence rate of ototoxicity was significantly lower in patients co-treated with LPZ compared to those without LPZ. These retrospective findings suggest that LPZ is also protective against CIO in humans. Taken together, co-treatment with LPZ may reduce the risk of CIO.

## Introduction

Cisplatin (CDDP), an inorganic platinum derivative, is one of the most widely used chemotherapeutic drugs against various cancers (Ghosh, 2019). CDDP, however, often causes many adverse events such as ototoxicity, nephrotoxicity, neuropathy, severe nausea and vomiting, and hematological toxicity (Ghosh, 2019). Specifically, hearing impairment is observed in over 60% of patients receiving chemotherapies with CDDP, which limits its clinical application (Karasawa and Steyger, 2015). Hearing impairment can lead to a decrease in quality of life, the development of depression, and poor communication abilities (Li et al., 2014; Nordvik et al., 2018; Bass et al., 2020). There remains an urgent need to develop strategies against CDDP-induced ototoxicity (CIO) (Lanvers-Kaminsky et al., 2017; Freyer et al., 2020; Santos et al., 2020; Yu et al., 2020; Pasquariello et al., 2021).

CDDP is accumulated in the stria vascularis of the cochlea and retained in the cochlea for a long time in humans, resulting in permanent hearing loss (Breglio et al., 2017). Organic cation transporter 2 (OCT2), also known as Solute Carrier Family 22 Member 2 (SLC22A2), is responsible for CDDP-induced ototoxicity via CDDP accumulation in the inner ear cochlea (Lanvers-Kaminsky et al., 2017). The single-nucleotide polymorphism rs316019 in *OCT2* (c.808G > T; Ser270Ala), which adversely affects the function of OCT2 (Frenzel et al., 2019), is associated with the protection against CIO (Lanvers-Kaminsky et al., 2015; Langer et al., 2020). CIO is not observed in *Oct2* knock-out (KO) mice (Ciarimboli et al., 2010). OCT2 is also involved in CDDP-induced nephrotoxicity (CIN) (Filipski et al., 2009; McSweeney et al., 2021). Considering these findings, we hypothesized that drugs that can inhibit OCT2 may protect against CIO.

Several studies have demonstrated that proton pump inhibitors (PPIs), drugs clinically used to treat and/or prevent gastroesophageal reflux disease and peptic ulcers (Scarpignato et al., 2016; Maret-Ouda et al., 2020), can inhibit OCT2 (Ikemura et al., 2017a). PPIs inhibit OCT2-mediated transport of metformin, a substrate of OCT2 (Nies et al., 2011). PPIs are not the substrates of OCT2 (Nies et al., 2011). Although the underlying mechanisms of how PPIs inhibit OCT2 remain unknown, several studies have demonstrated the protective role of PPIs against CIN. Lansoprazole (LPZ), a clinically used PPI, suppresses CIN through the inhibition of renal OCT2 in rats (Hiramatsu et al., 2020). The incidence rate of CIN is significantly lower in patients co-treated with LPZ compared to those without LPZ (Ikemura et al., 2017b). Pantoprazole, another PPI, also suppresses CIN in humans (Ghonaim et al., 2021). Therefore, we examined the effect of PPIs against CIO using *in vivo* fluorescence imaging of auditory hair cells in zebrafish and retrospective analyses using a public database, the Food and Drug Administration Adverse Event Reporting System (FAERS), and a database of electronic health records (EHRs) of patients in Mie University Hospital.

## Materials and Methods

### Ethics Statement

All animal experiments examined in the present study were approved by the Mie University Review Board for Animal Investigation (no. 2020–19) and conformed to the ethical guidelines established by the committee. The clinical study was conducted in accordance with the Declaration of Helsinki and was approved by the Ethics Committee of Mie University Graduate School of Medicine and Faculty of Medicine (no. 2021–170). Informed consent was obtained from participants through an opt-out method, because the data were collected retrospectively from electronic medical records.

### Materials

Yo-Pro-1 (Catalog number: Y3603) was purchased from Invitrogen (Tokyo, Japan). CDDP, cimetidine (CMD), and LPZ were purchased from Tokyo Chemical Industry Co., Ltd (Tokyo, Japan). Stock solutions of Yo-Pro-1 and LPZ were prepared by dissolving in dimethyl sulfoxide. Other chemicals were dissolved in 0.3 × Danieau’s solution (19.3 mM NaCl, 0.23 mM KCl, 0.13 mM MgSO_4_, 0.2 mM Ca(NO_3_)_2_, and 1.7 mM HEPES, pH 7.2). These solutions were diluted in 0.3 × Danieau’s solution to obtain the indicated concentrations. Lissamine-labeled control morpholino with no known target gene was purchased from Gene Tools (Philomath, OR, United States) and diluted with distilled water to prepare the stock solution.

### Zebrafish Husbandry

Zebrafish were maintained as described previously (Nishimura et al., 2016). Briefly, zebrafish were raised at 28.5 ± 0.5°C with a 14 h/10 h light/dark cycle. Embryos were obtained via natural mating and cultured in 0.3 × Danieau’s solution.

### Generation of *oct2* Crispant Zebrafish

There are two *oct2* genes (*zgc:64,076* and *zgc:175,176*) in zebrafish according to the database of genetic and genomic data specialized for zebrafish (https://zfin.org/). CRISPR RNA (crRNA) targeting *zgc:64,076* or *zgc:175,176*, trans-activating crRNA (tracrRNA), and recombinant Cas9 protein were obtained from FASMAC (Kanagawa, Japan) (sequences shown in Supplementary Table S1). Crispant zebrafish for *zgc:64,076* or *zgc:175,176* were generated according to methods described previously (Adachi et al., 2021). Briefly, the larvae exhibiting bright fluorescence of lissamine were selected at 1 day-post fertilization (dpf). Genomic DNA was extracted from zebrafish embryos injected with crRNA targeting *zgc:64,076* or *zgc:175,176* (crispants) or without crRNA (wild type, WT). Short fragments of the *zgc:64,076* and *zgc:175,176* gene encompassing the sites targeted by crRNAs were amplified from genomic DNA using the primers shown in Supplementary Table S1. Three-step PCR was carried out, followed by electrophoresis in 10% polyacrylamide gels (FUJIFILM Wako Pure Chemical, Osaka, Japan) and visualization by ethidium bromide staining.

### Drug Exposure in Zebrafish

Several studies have demonstrated that the loss of sensory hair cells can be caused by CDDP exposure within 4 h at 1,000 μM and 24 h at 50 μM (Ou et al., 2007; Domarecka et al., 2020). In this study, we wanted to analyze the effects of protectants at relatively early stages of CIO. Zebrafish larvae at 5 dpf were incubated in 0.3× Danieau’s solution containing CDDP (0, 50, 100, 250 μM, 500 μM, or 1,000 μM) alone for 2 h or co-treated with CDDP (0 or 250 μM) and CMD (0 or 1 mM) or LPZ (0, 0.1, 0.25, or 0.5 μM) for 2 h.

### *In Vivo* Imaging of Zebrafish Hair Cells

Sensory hair cells of zebrafish areanalogous to those of mammals (Lush and Piotrowski, 2014). Sensory hair cells express purinergic receptor P2X ligand-gated ion channel 7 (P2X7), which is activated by ATP secreted from supporting cells under physiological conditions (Zhao et al., 2005). Yo-Pro-1, a fluorescent cyanine dye, can permeate the cells via activated P2X7 (Virginio et al., 1999). Therefore, sensory hair cells of zebrafish can be visualized by vital staining with Yo-Pro-1 (Santos et al., 2006; Sasagawa et al., 2016). In this study, zebrafish larvae at 5 dpf were immersed in medium containing 2 μM of Yo-Pro-1 for 30 min. After staining, zebrafish were transferred to 0.3 × Danieau’s solution containing 2-phenoxyethanol (500 ppm) to be anesthetized and then transferred to glass slides. A few drops of 3% low-melting agarose solution were placed over the larvae, and the zebrafish were immediately oriented sideways dorsal-side-up. The zebrafish were then observed using an epifluorescence microscope (SZX7, Olympus, Tokyo, Japan) with the SZX-MGFP filter (Ex 475/30 nm, Em 510 nm). The 16-bit fluorescence images were converted to 256-gray-scale (0–255) images in Fiji (Schindelin et al., 2012). Considering the previous studies (Chiu et al., 2008; Sasagawa et al., 2016), we measured the fluorescence signal of three neuromasts (MI1, MI2, and O2) (Raible and Kruse, 2000). Neuromasts are composed of different types of cells, including hair cells and supporting cells, which are important for hearing and balance in zebrafish (Froehlicher et al., 2009). The sum of the fluorescent areas of these neuromasts (shown in small rectangles in each figure) with signals over the threshold (25 in the 256-gray scale) was calculated and reported as the fluorescent area of hair cells in a zebrafish.

### qPCR Analysis

Zebrafish were harvested at 5 dpf and stored in RNA-later (Applied Biosystems, Foster City, CA, United States). Total RNA was extracted using an RNeasy Mini kit (Qiagen, Germantown, MD, United States) according to the manufacturer’s protocol. cDNA was generated using a ReverTra Ace qPCR RT Kit (Toyobo, Osaka, Japan) and qPCR was performed using QuantStudio3 real-time PCR machine (Applied Biosystems, Thermo Fisher, United States) with THUNDERBIRD SYBR qPCR Mix (Toyobo). The thermal cycling conditions were 95°C for 60 s, followed by 40 cycles of 95 °C for 15 s, 60°C for 15 s, and 72°C for 45 s. Expression of *zgc:64,076* and *zgc:175,176* mRNA was normalized to that of the eukaryotic translation elongation factor 1 alpha 2 (*ef1a*) to correct for variability in the initial template concentration and reverse transcription efficiency. qPCR primer sequences are shown in Supplementary Table S1.

### Retrospective Analysis Using the FAERS Database

Data on patient demographics and administration information, drug/biologic information, and adverse events from July 2014 to December 2020 were obtained from the FAERS database. Duplicate reports were excluded in accordance with the FDA recommendations. Data analyses were performed using ACCESS^®^ 2019 software (Microsoft, Redmond, WA). Data associated with CDDP administration were extracted. Disease names were defined using the Medical Dictionary for Regulatory Activities (MedDRA/J) version 24.0. The standardized MedDRA Query (SMQ) was used for searching for hearing impairment (SMQ code: 20,000,171). The effect of PPIs on CDDP-associated hearing impairment was evaluated using the reporting odds ratio (ROR). To calculate the ROR, CDDP-associated hearing impairment and all other reported adverse events associated with CDDP were defined as “cases” and “non-cases,” respectively. The RORs were calculated from two-by-two contingency tables of counts with or without PPI. RORs were expressed as point estimates with 95% confidence interval (CI). Although stratification based on age, gender, and primary diseases can be done using the FAERS database (Nagashima et al., 2016; Hosoya et al., 2022), we did not perform the stratified analysis due to the small number of the CIO cases treated with PPIs.

### Retrospective Analysis Using EHRs of Mie University Hospital

Clinical data were collected from EHRs of patients who received CDDP in the Department of Otorhinolaryngology-Head and Neck Surgery or the Department of Medical Oncology of Mie University Hospital during January 2010 and April 2021. Among 463 hospitalized adult patients treated with CDDP, 340 patients treated with ≥300 mg of total CDDP dose for cancers in the lung, esophagus, stomach, kidney, mammary glands, thymus, and reproductive tissues were enrolled in this study. Patients were excluded if they were defined as having hearing loss before CDDP treatment (*n* = 5), ear disease (*n* = 6), prescribed aminoglycoside antibiotics (*n* = 1), and PPIs prescribed other than LPZ (*n* = 39). Ototoxicity was defined as hearing loss, sensorineural hearing loss, or tinnitus following CDDP treatment.

### Statistical Analyses

The results are expressed as the mean ± standard deviation. Differences between two groups were assessed using unpaired t-tests. Statistical comparisons for multiple groups were performed using one-way analysis of variance followed by Dunnett’s test or Tukey’s multiple comparison test. Statistical comparisons between two groups in clinical data were performed using the Mann-Whitney *U* test and Fisher’s exact test for continuous and categorical variables, respectively. All statistical analyses were performed using GraphPad Prism 9 (GraphPad Software Inc. San Diego, CA). The significance was established at a *p* value < 0.05.

## Results

### LPZ Ameliorated the Decrease of Fluorescence Signals in Hair Cells Caused by CDDP

**FIGURE 1:**
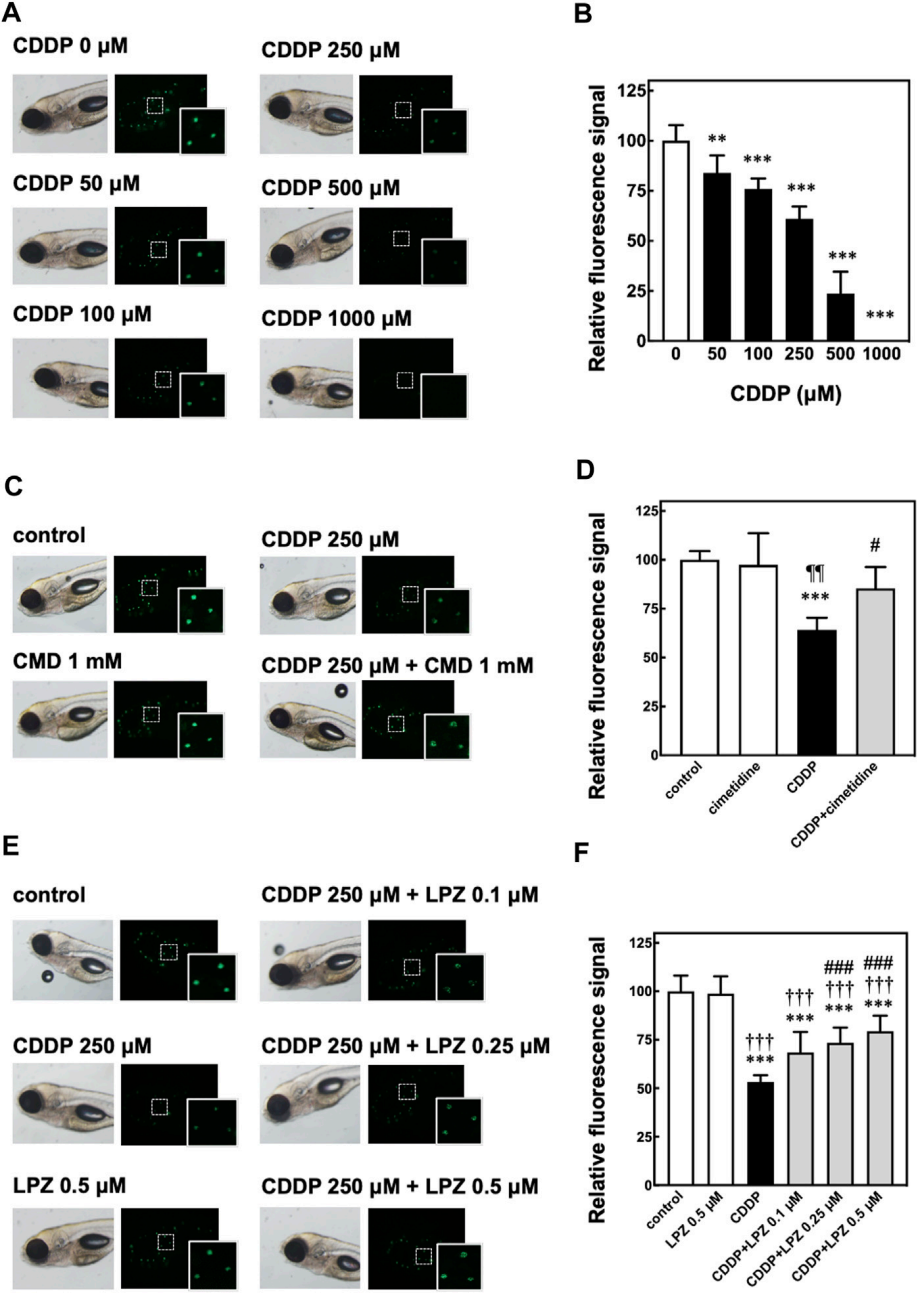
*In vivo* fluorescence imaging of hair cells in zebrafish to assess CDDP-induced ototoxicity and otoprotective effects of cimetidine and lansoprazole. **(A,B)** Zebrafish were treated with CDDP at indicated doses for 2 h, followed by staining with Yo-Pro-1 of sensory hair cells. The fluorescence signals of three neuromasts including hair cells shown in the white rectangles were calculated and are shown as the percentage of the signal in zebrafish without CDDP treatment. **(C–F)** Zebrafish were treated with or without cimetidine (CMD, **(C,D)** or lansoprazole (LPZ, **(E,F)** to examine their effect on the decrease of fluorescence signals in hair cells in zebrafish exposed to CDDP. Quantitative analyses were performed as described above.

We first evaluated the ototoxicity of CDDP in zebrafish by *in vivo* fluorescence imaging of sensory hair cells stained with Yo-Pro-1. The *in vivo* imaging revealed that the fluorescence signals of hair cells in zebrafish were decreased dependent on the dose of CDDP (Figures 1A,B). Because the fluorescence of hair cells in zebrafish treated with 250 μM CDDP was around 50% (Figure 1B), we selected this dose to examine the otoprotective effects of CMD and LPZ against the ototoxicity of CDDP.

Previous studies have demonstrated that CMD inhibits OCT2 and suppresses both CIO and CIN in rodents (Okuda et al., 1996; Ciarimboli et al., 2010). CMD is a substrate of OCT2 and works as a competitive inhibitor of other OCT2 substrates (Urakami et al., 1998; Harper and Wright, 2013; Yin et al., 2016). Consistent with these studies, co-treatment with 1 mM of CMD significantly suppressed the decrease of fluorescence signals in hair cells in zebrafish treated with 250 μM of CDDP (Figures 1C,D).

We then examined the effect of LPZ on the decrease of fluorescence signals in hair cells in zebrafish treated with 250 μM of CDDP. As shown in Figures 1D,E, LPZ dose-dependently inhibited the decrease of fluorescence signals in hair cells in zebrafish treated with 250 μM of CDDP. These results suggest that LPZ ameliorates CIO through inhibition of OCT2.

### CDDP Caused Hair Cell Damage Through oct2 in Zebrafish

**FIGURE 2:**
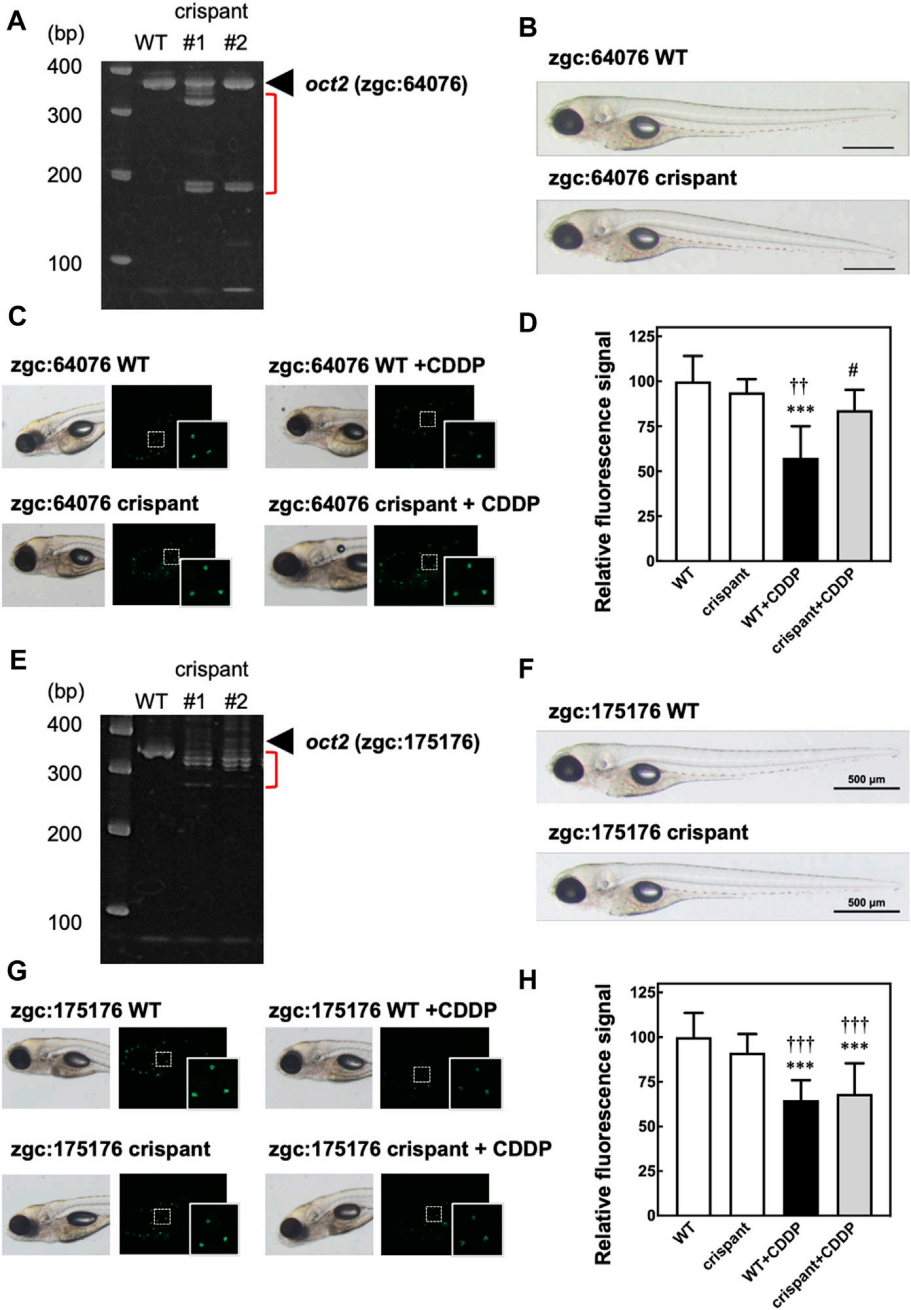
*In vivo* fluorescence imaging of hair cells in crispant zebrafish to assess the involvement of oct2 in the decrease of fluorescence signals caused by CDDP. Crispant zebrafish with knockout (KO) of *zgc:64,076* or *zgc:175,176*, zebrafish homologs of OCT2, were generated using CRISPR-Cas9 technology. **(A,E)** Electrophoresis of genomic PCR around the sequences targeted by genome editing of *zgc:64,076* **(A)** or *zgc:175,176* **(E)**. **(B,F)** Bright field imaging of both wild-type (WT) and crispant zebrafish for zgc:64,076 **(B)** and zgc:175,176 **(F)**. C, D, G, and **(H)** WT and crispant zebrafish for *zgc:64,076* **(C,D)** and *zgc:175,176* **(G,H)** were treated with or without CDDP, followed by staining with Yo-Pro-1 of sensory hair cells. The fluorescence signals of three neuromasts including hair cells shown in the white rectangles were calculated and are shown as the percentage of the signal of WT zebrafish without CDDP treatment.

To examine the role of oct2 in CIO in zebrafish, we generated two crispant zebrafish with KO of *zgc:64,076* or *zgc:175,176*, zebrafish homologs of *OCT2*. To KO *zgc:64,076*, we designed crRNA targeting the sequence including the protospacer adjacent motif located behind the start codon in the first exon, and then injected the crRNA with tracrRNA and Cas9 protein into zebrafish embryos. At 1 dpf, the genomes were extracted, and PCR analysis was performed using primers to amplify the genomic regions around the sequences targeted by the crRNA. As shown in Figure 2A, extra bands (shown in red bracket) were observed below the main PCR products (black arrowhead) in the crispant but not WT zebrafish, suggesting that KO of *zgc:64,076* were generated in the crispant zebrafish. There were no external abnormalities in the *zgc:64,076* crispant zebrafish (Figure 2B). *In vivo* fluorescence imaging of zebrafish hair cells revealed that CDDP treatment caused a decrease of fluorescence signals in hair cells in WT but not *zgc:64,076* crispant zebrafish (Figures 2C,D).

We also generated *zgc:175,176* crispant zebrafish using CRISPR-Cas9 technology. Genomic PCR analysis confirmed KO of *zgc:175,176* in the crispant zebrafish (Figure 2E). No external abnormalities were observed in the *zgc:175,176* crispant zebrafish (Figure 2F). *In vivo* fluorescence imaging of zebrafish hair cells, however, revealed that CDDP treatment caused a decrease in fluorescence signals in hair cells in both WT and *zgc:175,176* crispant zebrafish (Figures 2G,H). These results are consistent with the result showing that the expression of *zgc:64,076* was significantly higher than that of *zgc:175,176* in zebrafish at 5 dpf (Supplementary Figure S1) and suggest that *zgc:64,076* is mainly involved in the CDDP-induced hair cell damage.

### The Incidence Rate of CIO Was Decreased in Patients Treated With LPZ

To examine the effect of LPZ on the incidence of CIO in humans, we analyzed the FAERS database that has been successfully used for drug repositioning (Zamami et al., 2021). We extracted 29,976 patients who experienced ototoxicity in relation to CDDP treatment. We then analyzed the reporting ratio of CIO, the ROR, and the 95% CI (Table 1). The reporting ratio of CIO in patients with LPZ (1.0%) was significantly lower than that in patients without LPZ (2.7%, *p* = 0.039). The ROR of CIO in patients treated with LPZ was 0.37 (95% CI: 0.14–0.99). In addition, the reporting ratio of CIO in patients treated with PPIs other than LPZ (i.e., omeprazole and esomeprazole) was also significantly lower than in that in patients treated without these PPIs (Table 1), suggesting that the protective effects of LPZ against CIO may be a class effect of PPIs.

**TABLE 1:** Effect of PPIs on the incidence of CDDP-induced ototoxicity in FAERS.

PPIs	CDDP-Induced Ototoxicity		ROR	*p* value
	Without	With	(95%CI)	
Lansoprazole	801/29,586 (2.7)	4/390 (1.0)	0.37 (0.14–0.99)	0.039
Dexlansoprazole	805/29,938 (2.7)	0/38 (0)	0.47 (0.02–7.65)	0.627
Rabeprazole	805/29,911 (2.7)	0/65 (0)	0.28 (0.02–4.46)	0.424
Esomeprazole	802/29,618 (2.7)	3/358 (0.8)	0.30 (0.10–0.95)	0.030
Omeprazole	804/29,347 (2.7)	1/629 (0.2)	0.06 (0.01–0.40)	<0.001
Pantoprazole	791/29,916 (2.7)	14/860 (1.6)	0.59 (0.34–1.01)	0.053

The incidence rate of CDDP-induced ototoxicity was presented as cases/(cases + non-cases) (%).

We then validated the effect of LPZ on the incidence of CIO using the EHR of Mie University Hospital. According to the inclusion and exclusion criteria, we enrolled 289 patients in this study. Table 2 shows a comparison of patient characteristics between patients with ototoxicity and without ototoxicity. The rate of concomitant LPZ in patients without ototoxicity (88 out of 260 patients, 34%) was significantly higher than that in patients with ototoxicity (2 out of 29 patients, 7%, *p* = 0.002). Taken together, these results suggest that LPZ may be protective against CIO in humans.

**TABLE 2:** Comparison of characteristics between CDDP-treated patients with ototoxicity and without ototoxicity in EHR of Mie University Hospital.

Patient Characteristics	Without Ototoxicity (*n* = 260)	With Ototoxicity (*n* = 29)	*p* value
Male	213 (82%)	21 (72%)	0.217
Age (years)	64 [20—81]	61 [41—80]	0.128
Body weight (kg)	51.5 [33.4—83.4]	49.0 [35.8—64.2]	0.991
Body surface area (m^2^)	1.540 [1.265—2.006]	1.581 [1.258—1.772]	0.659
Total CDDP dose (mg)	668 [300—2,334]	763 [390—1,380]	0.145
LPZ	88 (34%)	2 (7%)	0.002
Baseline biological parameters			
eGFR (ml/min)	80.6 [42.8—124.3]	81.9 [59.0—104.9]	0.833
Scr (mg/dl)	0.73 [0.52—1.46]	0.72 [0.44—0.94]	0.331
BUN (mg/dl)	13 [5—45]	13 [7—31]	0.246
AST (U/L)	22 [7—92]	23 [14—72]	0.189
ALT (U/L)	17 [5—84]	17 [7—109]	0.391
γ-GTP (U/L)	31 [10—579]	37 [11—127]	0.122

Values are presented as median [range] or number (%). ALT: alanine transaminase, AST: aspartate transaminase, BMI: body mass index, BUN: blood urea nitrogen, CDDP: cisplatin, GFR: estimated glomerular filtration rate, Scr: serum creatinine, γ-GTP: gamma-glutamyl transpeptidase.

## Discussion

In this study, we demonstrated that co-treatment with LPZ or KO of *zgc:64,076*, a homolog of *OCT2*, suppressed the decrease of fluorescence signals in sensory hair cells in zebrafish treated with CDDP. We also found that the incidence rate of CIO was significantly lower in patients treated with LPZ compared to those without LPZ.

The inhibitory effect of PPIs on OCT2 and the protective effects of PPIs against CIN have been demonstrated in several studies (Nies et al., 2011; Hacker et al., 2015; Ikemura et al., 2017b; Gao et al., 2019; Gao et al., 2020; Hiramatsu et al., 2020; Ismail et al., 2020; Ghonaim et al., 2021; McSweeney et al., 2021). A variant of human *OCT2* rs316019, which inhibits the function of OCT2 (Frenzel et al., 2019), is associated with protection against both CIN (Iwata et al., 2012) and CIO (Lanvers-Kaminsky et al., 2015; Langer et al., 2020). KO of *Oct2* is also protective against CIO (Ciarimboli et al., 2010). These findings suggest that inhibition of OCT2 is a promising approach against both CIN and CIO (Lanvers-Kaminsky et al., 2017; Gessner et al., 2019; McSweeney et al., 2021; Pasquariello et al., 2021). Both PPIs and CMD can inhibit multidrug and toxin extrusion 1 (MATE1), which is a key transporter for the excretion of CDDP from renal tubular epithelial cells (Nakamura et al., 2010; Yin et al., 2016; Guo et al., 2018). However, PPIs are not the substrates of OCT2, suggesting that the inhibition of MATE1 by PPIs may not have a significant impact on the excretion of CDDP (Nies et al., 2011; Hiramatsu et al., 2020). Consistent with this idea, the incidence of CIN in head and neck cancer patients treated with pantoprazole (PTZ), which is a PPI known to inhibit OCT2 (Nies et al., 2011; Hacker et al., 2015), is significantly lower in those without PTZ (Ghonaim et al., 2021). It should be noted, however, that PTZ is not protective against CIN and CIO in patients diagnosed with osteosarcoma treated with CDDP, methotrexate, and doxorubicin (Fox et al., 2018). Antioxidant signaling may also be involved in the protective effect of LPZ against CIO. CDDP generates oxidative stress which leads to apoptosis and necroptosis of auditory hair cells (Ramkumar et al., 2021). LPZ can exert antioxidant effects through activation of nuclear factor erythroid 2-related factor 2 (Takagi et al., 2009). Immunohistochemical analysis of zebrafish neuromasts using antibodies against hair cell markers, cleaved caspase 3, and annexin V (Shahab et al., 2021; Warchol et al., 2021) should be done to elucidate the protective mechanism of LPZ against CIO.

In this study, we found that the incidence of CIO in the FAERS database was significantly lower in patients treated with LPZ, omeprazole (OMZ), or esomeprazole, but not PTZ. Although these PPIs inhibit OCT2, there are several differences among them (Nies et al., 2011; Hacker et al., 2015). In human embryonic kidney cells (HEK293) stably overexpressing OCT2, OMZ inhibits the uptake of metformin, 4-(4-(dimethylamino)styryl)-N-methylpyridinium iodide (ASP+), and N-methyl-4-phenylpyridinium iodide (MPP+), which are substrates of OCT2 (Hacker et al., 2015). In the same cell line, LPZ inhibits the uptake of metformin and ASP+, whereas PTZ inhibits the uptake of metformin and MPP+ (Hacker et al., 2015). The IC_50_ of LPZ to OCT3 is smaller than that of LPZ to OCT2, whereas the IC_50s_ of OMZ and PTZ to OCT2 are smaller than those to OCT3 in HEK293 cells stably overexpressing one of these OCTs (Nies et al., 2011). These findings suggest that the inhibition of CDDP uptake into cells through OCT2 may differ among these PPIs.

This study has some limitations. First, the protective role of LPZ against the ototoxicity caused by CDDP at relatively low doses and long exposure remains to be elucidated. Second, the localization of *zgc:64,076* and *zgc:175,176* in zebrafish sensory hair cells is currently unknown. Third, crispants, CRISPR-generated F0 mutants, for *zgc:64,076* or *zgc:175,176* were used to examine the role of zebrafish *OCT2* homologs in CIO. Although crispant zebrafish have been successfully used to examine the functions of gene of interest (Watson et al., 2020; Kroll et al., 2021), a validation using homozygous germline mutants for the expression of zebrafish *OCT2* homologs and protection against CIO is important to strengthen the findings of this study. Fourth, although we demonstrated that the incidence rate of CIO was significantly lower in patients treated with LPZ compared to those treated without LPZ in the EHRs of Mie University Hospital, the number of patients with CIO (*n* = 29) was relatively low among the total number of patients (*n* = 260). Additional real-world evidence among general populations is required to confirm whether LPZ or other PPIs can protect against CIO (Rajan et al., 2022). Fifth, we were not able to analyze the effect of LPZ on the survival of patients treated with CDDP. Whether these PPIs negatively affect the uptake of CDDP into tumor cells and affect patient survival should be clarified (Sprowl et al., 2013; Kichenadasse et al., 2021).

To our knowledge, however, this is the first study to demonstrate a protective effect of LPZ against CIO, and warrants further investigation to clarify the mechanisms underlying this protection.

## Data Availability

The raw data supporting the conclusion of this article will be made available by the authors, without undue reservation.

## Abbreviations

ASP+: 4-(4-(dimethylamino)styryl)-N-methylpyridinium iodide; CDDP, Cisplatin; CMD, cimetidine; CI, confidence interval; CIN, CDDP-induced nephrotoxicity; CIO, CDDP-induced ototoxicity; crRNA, CRISPR RNA; dpf, day-post fertilization; EHRs, electronic health records; FAERS, the Food and Drug Administration Adverse Event Reporting System; KO, knock-out; LPZ, lansoprazole; MATE1, multidrug and toxin extrusion 1; MPP+: N-methyl-4-phenylpyridinium iodide; OCT2, organic cation transporter 2; ROR, reporting odds ratio.
