# Supplementary figures and images for: Repositioning of Lansoprazole as a Protective Agent Against Cisplatin-Induced Ototoxicity
Source: Front Pharmacol. 2022 Jul 15;13:896760. doi: 10.3389/fphar.2022.896760 (PMC9336179; doi:10.3389/fphar.2022.896760)

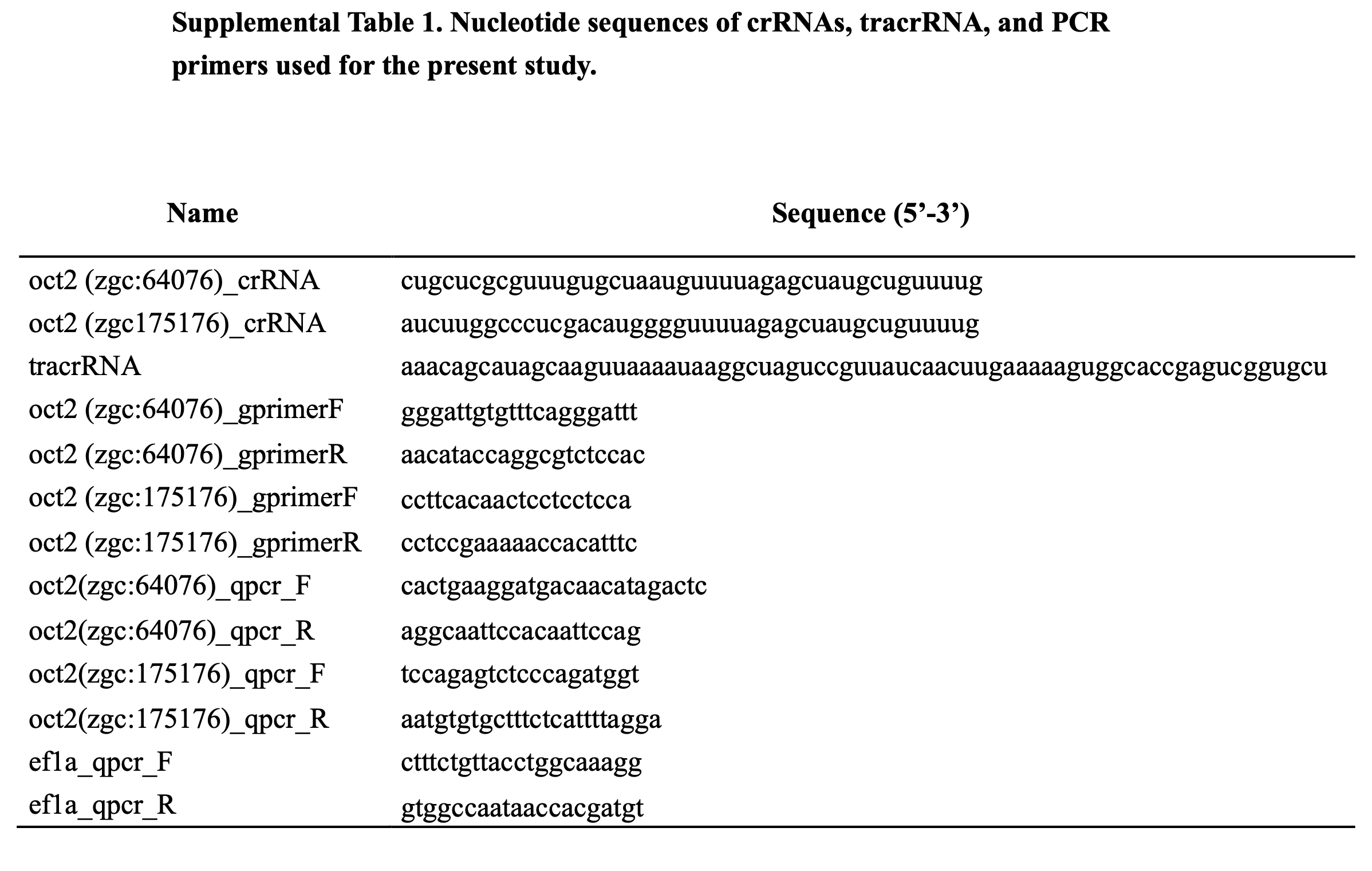

Supplement: Supplementary file 1 [file Image2.TIF]

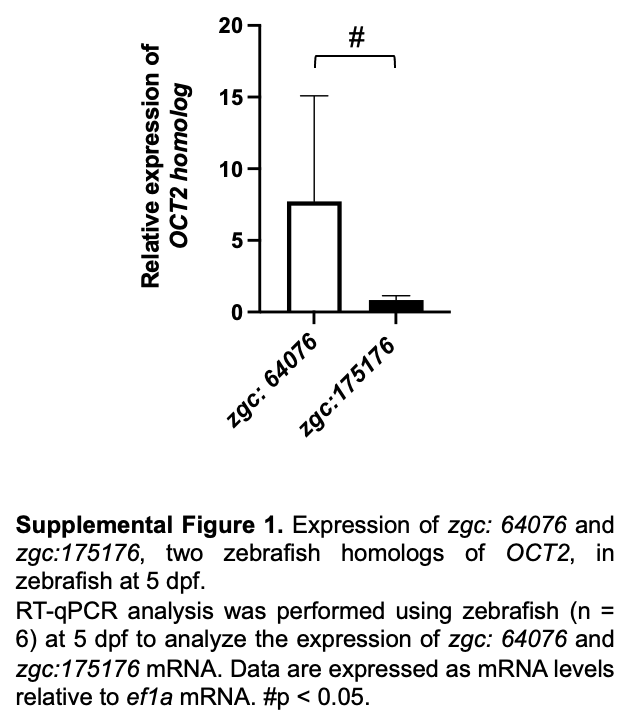

Supplement: Supplementary file 2 [file Image1.TIF]
